# Preliminary Feasibility Investigation on Reutilization of Recycled Crushed Clay Bricks from Construction and Demolition Waste for Cement-Stabilized Macadam

**DOI:** 10.3390/ma15093171

**Published:** 2022-04-27

**Authors:** Dongxing Wu, Wenchao Chu, Longlin Wang, Wensheng Wang, Haoyun Wang, Xuanhao Shangguan, Xiang Cui

**Affiliations:** 1 First Detachment, Guangxi Transportation Comprehensive Administrative Law Enforcement Bureau, Nanning 530007, China; wudx_gx@163.com; 2 China State Construction Railway Investment & Engineering Group Co., Ltd, Beijing 100053, China; luogbjlu@163.com; 3 Bridge Engineering Research Institute, Guangxi Transportation Science and Technology Group Co., Ltd., Nanning 530007, China; 4 School of Civil Engineering, Southeast University, Nanjing 211189, China; 5 College of Transportation, Jilin University, Changchun 130025, China; wanghy1717@mails.jlu.edu.cn (H.W.); sgxh1719@mails.jlu.edu.cn (X.S.); 6 Lunan Technician College, Linyi 276000, China; wanghjlu@163.com

**Keywords:** cement-stabilized macadam, recycled clay brick, mechanical performances, durability, shrinkage characteristics, leaching toxicity

## Abstract

Utilizing recycled crushed clay brick (RCB) from C&D waste in road engineering construction as the substitute for natural aggregates has attracted a lot of attention, which would be a promising step forward towards sustainable development and green construction. The objective of this study is to assess the feasibility of cement-stabilized macadam (CSM), incorporating various RCB fine aggregate substitution ratios. For this purpose, the physical and chemical properties of RCB fine aggregate was tested, and RCB exhibited a porous surface micro-morphology, high water absorption and pozzolanic activity. Subsequently, a comprehensive experimental investigation of modified CSM with RCB has been carried out based on laboratory tests concerning the mechanical and shrinkage properties. Results showed that higher RCB fine aggregate substitution ratio resulted in lower unconfined compressive strength, and the negative influence of RCB on unconfined compressive strength would decrease gradually, varying curing time; however, the higher the RCB substitution ratio was, the larger the indirect tensile strength at 90 d curing time of the late curing period was. CSM containing RCB had an overall increasing accumulative water loss rate, accumulative strain of dry shrinkage and average coefficient of dry shrinkage, except that 20% RCB resulted in an excellent dry shrinkage property. Moreover, RCB with pozzolanic activity reacted very slowly mainly at later ages, enhancing the interfacial transition zone.

## 1. Introduction

The generation of construction and demolition (C&D) waste, such as clay brick, concrete, etc., is being accelerated by construction of new and old urban areas and renovation of the urban–rural fringe [1,2]. It was reported that America produces 145 million tons of C&D waste annually [1], India produces about 14.5 million tons [3], while as much 1.8 billion tons was generated in 2017 in China [4]. As the leading country of world municipal solid waste generation (accounting for about 30% of the global total), an increasing volume of C&D waste accounts for one third of the total municipal solid waste in China; however, the ratio of recycling and reprocessing C&D waste is only around 5% [5]. Furthermore, according to the statistics, C&D waste was estimated to constitute to approximately 25~45% of the total solid waste in landfill [6]. Bricks, as a widely used construction material, are inevitably damaged during the reconstruction process, such as through demolition and construction activities. Over the past 50 years, approximately 20~30 billion clay bricks have been produced in China, which have been or will be turned into a huge amount of C&D waste [7].

Currently, a relatively complete set of reutilization technologies for clay bricks has been formed, in which the clay bricks from C&D waste are separated, screened, crushed and stripped of impurities to manufacture the recycled crushed clay brick (RCB) [8,9]. However, based on the consideration of environmental protection and resource conservation including carbon emissions reduction, the mining of natural aggregates has been banned in many areas of China. There is an imbalance between the supply and demand of aggregates as well as an increasingly prominent contradiction between natural aggregate consumption and C&D waste disposal. Some studies have investigated RCB reutilization as a partial cement substitute due to its pozzolanic activity or coarse and fine aggregate replacements [10]. It has been observed that it is feasible to reuse RCB as a partial substitute in cementitious construction materials. Although it may slightly reduce the mechanical and durability properties, the experimental results indicated that adding RCB could still meet application requirements [11]. Furthermore, recycled aggregates have been used as coarse or fine aggregates for pavement bases or subbases in about 38 states of America [12]. Semi-rigid base has been widely used for road engineering due to its higher strength, better loading distribution and excellent wholeness over a period of decades. Its main materials generally include cement-stabilized macadam (CSM), lime–fly-ash-stabilized macadam, cement–fly-ash-stabilized macadam and lime-stabilized macadam, etc. [13], and the semi-rigid base is becoming the predominant type of base and subbase for highways and urban roads, especially in China [14,15]. CSM, as one of the main semi-rigid base materials, is a kind of composite construction material composed of proper aggregate gradation, 3~8% cement of aggregate by weight as well as optimum content of water [16]. The potential of CSM incorporating RCB is worth studying in order to improve the application of CSM and supply an opportunity to use RCB from C&D waste, which is widely accepted as a cost-effective reutilization means [12,17].

Previous studies have attempted to explore the application of RCB as aggregate for cement-based material. The comparative study of Arulrajah et al. reported that about 25% RCB could be satisfactorily added to recycled aggregates in the application of pavement subbase, and the RCB content has marginal effects on the mechanical properties and relatively obvious influences on dry density as well as moisture content of CSM [18]. Data from several studies suggested that the substitution ratio of recycled aggregates in pavement materials should not exceed up to 80% [19,20]. Remarkably, a great deal of studies have investigated the effect of RCB as coarse or fine aggregate on cement-based materials. It has been conclusively proven that RCB replaces coarse aggregate only partially, while the replacement rate of RCB for fine aggregate can be as high as 100%. Generally, there will be a reduction in the mechanical properties of cement-based materials by replacing coarse aggregate with RCB. The lower inherent strength of RCB may be considered as one of the major factors for the strength reduction of cement-based materials due to the main skeleton component of coarse aggregate [21]. Nevertheless, several studies have reported that the replacement rate of coarse aggregate by RCB could be up to 50% in cement-based material, which still meets the application requirements and the RCB replacement rate within 20% would have no prominent negative influence on cement-based material [1,22]. Unlike coarse aggregate replacement, the possibilities of applying RCB fully instead of fine aggregate in cement-based materials have been investigated, and the clear evidence from experimental observations supported the full utilization of RCB replacement and the benefits of RCB fine aggregate for the machinal and shrinkage properties [23,24]. It has been demonstrated that a lower drying shrinkage was measured for cement-based material with 20% RCB replacing fine aggregate, which may be due to the restraining effect of RCB and the internal curing effect [23]. In addition, the findings of these research confirmed that the incorporation of RCB with pozzolanic activity into cement-based material can inhibit alkali silica reactions [25]. Consequently, the active pozzolanic materials in RCB aggregates would react with the hydration product (calcium hydroxide, Ca(OH)_2_), generating the hydrated calcium silicate (C-S-H), which can lead to an expansion in cement-based material [24]. The benefits of RCB aggregate, therefore, may be driven by pozzolanic action provided by fine RCB aggregate.

The purpose of this paper is to explore the feasibility and mechanism analysis of cement stabilized macadam with RCB as a fine aggregate. In this paper, RCB fine aggregate, crushed by jaw crusher, was selected and its physical and chemical properties analyzed by energy dispersive spectroscopy (EDS) and X-ray diffraction (XRD) technologies. Unconfined compressive strength, indirect tensile strength and dry shrinkage of modified CSM incorporating various RCB fine aggregate substitution ratios were tested to evaluate the mechanical and shrinkage properties. Finally, the pozzolanic activity was also tested for RCB using chemical detection methods for a further understanding of the strength mechanism of modified CSM.

## 2. Raw Materials and Methods

### 2.1. Raw Materials

The cement adopted is rapid-hardening ordinary silicate cement (P.O 42.5R), and its setting time and strength of P.O 42.5R were tested, which could satisfy regulations of standards (GB175-2007, JTG/T F20-2015). In this study, 5% of cement dosage in mixtures is used in the base course of pavement. The type of natural aggregates used for CSM in this study is natural crushed basalt aggregate (BA). The RCB was collected from the construction waste in a shantytown demolition site near Changchun, China. First, construction waste collected from C&D were treated to clean up sundries such as waste wire, wooden frame, glass, and so on. Second, recycled waste clay bricks were crushed into particles using an impact jaw crusher, and the maximum size was controlled, set to 31.5 mm. Finally, those RCB were cleaned and sieved. In the case of large-scale implementation, the classification and screening equipment for C&D waste is suggested to be adopted for the material selection. Their apparent specific gravity, water absorption, needle particles, crushing values, liquid limit and plastic limit were tested. Technical indicators are summarized in Table 1, which comply with Chinese standards (JTG/T F20-2015).

**Figure 1:**
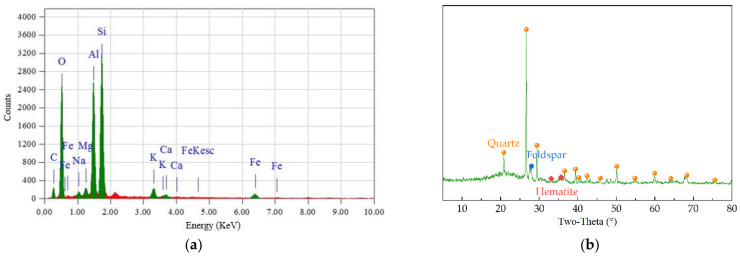
Properties of RCB aggregate: (**a**) EDS spectrum; (**b**) XRD pattern.

It can be found from EDS spectrum analysis in Figure 1a that RCB contains a lot of oxygen, silicon, aluminum, calcium, carbon, iron, magnesium and other elements. There are many oxide forms in RCB due to the high oxygen element content, which would provide some potential activity. The porous surface micro-morphology and high oxides are responsible for larger water absorption and crushed value [4,26]. Figure 1b shows the mineral compositions of RCB aggregate by the XRD pattern. There are several significant peaks representing inorganic crystalline phase of quartz (SiO_2_), a small amount of weak peaks representing the crystalline phase of feldspar (K_2_Al_2_Si_6_O_6_), and hematite (α-Fe_2_O_3_) in the XRD pattern, in which the quartz (SiO_2_) is the major crystalline phase. Based on its physical along with chemical properties, RCB aggregate has potential of pozzolanic reaction and cementitious activity [24,27].

### 2.2. Gradation Design and Samples Preparation

#### 2.2.1. Aggregate Gradation

**Figure 2:**
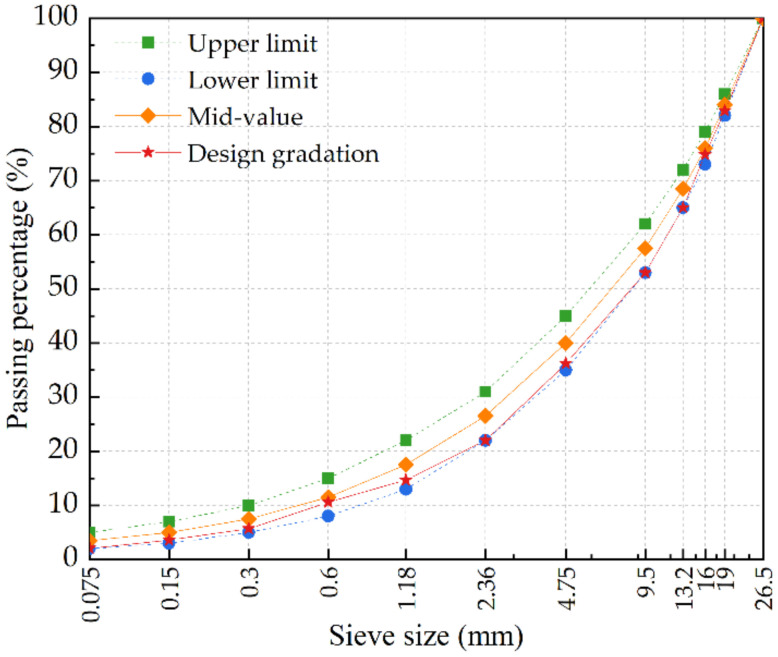
Aggregate gradation curve of CSM.

The difference of aggregate gradation is the main reason for the diversity of mechanical and shrinkage properties of CSM. Referring to Chinese standard (JTG/T F20-2015), the aggregate gradation range and CSM gradation curve are illustrated in Figure 2, based on our previous research results and engineering experience [28]. Considering that RCB with large water absorption, low density as well as soft particles, the BA (≤4.75 mm) was substituted by RCB at six ratios of 0%, 20%, 40%, 50%, 60%, 80%, named CSM-BA, CSM-RCB20, CSM-RCB40, CSM-RCB50, CSM-RCB60 and CSM-RCB80, respectively.

#### 2.2.2. Mixture Design and Samples Preparation

**Figure 3:**
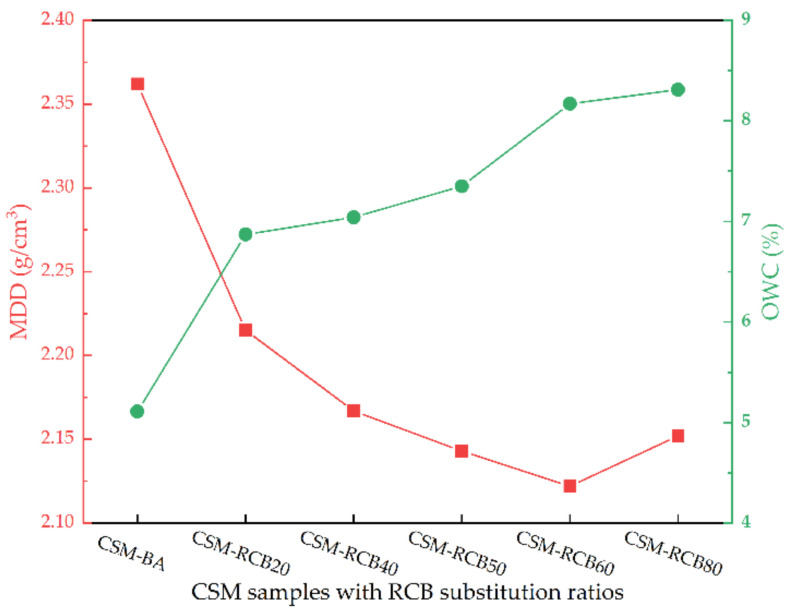
MDD and OWC results of CSM samples.

Referring to the Class C heavy compaction test method T0804-1994 in Chinese standard (JTG E51-2009), optimum water content (OWC) and maximum dry density (MDD) for CSM containing various RCB substitutions could be determined. According to the aggregate gradation structure specified in Figure 2 and 5% cement dosage of CSM, the Proctor standard compaction tests under six grades of water content with an interval of 0.5% were carried out. The thoroughly blended aggregates and cement were poured into the cylindrical compactor in three layers at 98 times of hammers per layer. According to the obtained law for dry density along with water content from compaction test, OWC and MDD of CSM samples have been obtained using quadratic polynomial fit method, summarized in Figure 3. With RCB substitution ratio in CSM, the MDD of CSM decreased gradually, while the OWC showed an increasing trend. This result may be explained by the fact that compared to BA, RCB is provided with larger water absorption as well as lower density (as listed in Table 1).

According to the above-obtained MDD and OWC of CSM samples with different substitution ratios of RCB, the CSM samples were prepared with 98% compactness by the static compaction in Chinese standard (JTG E51-2009). Referring to T0843-2009 in JTG E51-2009, a series of cylindrical samples (ϕ150 mm × 150 mm) were made to measure unconfined compressive strength and indirect tensile strength. Following T0844-2009 in Chinese standard (JTG E51-2009), cuboid beam samples (100 mm × 100 mm × 400 mm) were molded for testing dry shrinkage. After 2 min steady pressure for cylindrical samples or 5 min steady pressure for cuboid beam samples, the applied pressure was unloaded, and the test mold was removed. The compacted samples were demolded after 4~6 h for cylindrical samples or 24 h for cuboid beam samples and were stored carefully in sealed plastic bags and cured at a typical controlled condition (20 ± 2) °C as well as RH > 95%, referring to T0845-2009 in the Chinese standard (JTG E51-2009).

### 2.3. Experimental Methods

**Figure 4:**
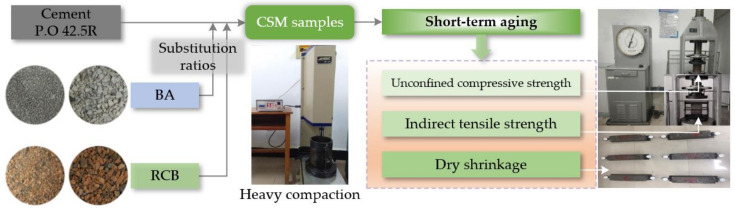
The flowchart of this study.

The CSM samples were firstly prepared with different substitution ratios of RCB, and then the CSM samples could be used for unconfined compressive strength test, indirect tensile strength test and dry-shrinkage test. The flowchart with illustrations of the experimental study is shown for describing the research phases in Figure 4.

#### 2.3.1. Unconfined Compressive Strength

Unconfined compressive strength tests were carried out for scraped cylindrical CSM samples (after curing for 7 d, 28 d, 90 d, 180 d) through 2000 KN hydraulic pressure universal testing machine in accordance with T0805-1994 in Chinese standard (JTG E51-2009), as demonstrated in Figure 4. The cylindrical sample and spherical support were placed at the center of the vertical load and loaded at the rate of 1 mm/min until damage, meanwhile recording its maximum load (*P_C_*). Then unconfined compressive strength (*R_C_*) can be obtained by Equation (1).

(1) $$R_{C}=\frac{P_{C}}{A}=\frac{P_{C}}{\pi D^{2}},$$

where *A* is cross-sectional area (mm^2^), *D* is diameter for cylindrical samples (mm).

#### 2.3.2. Indirect Tensile Strength

In line with T0806-1994 in JTG E51-2009, indirect tensile strength tests were performed for cured cylindrical CSM samples (90 d) using a hydraulic pressure universal testing machine, as shown in Figure 4. The cylindrical sample was placed horizontally on the fixture to ensure that two battens were placed at both cross-section ends of the test sample and perpendicular to the lifting platform of the hydraulic pressure universal testing machine, then stable loading of 1 mm/min until failure. Indirect tensile strength (*R_T_*) could be calculated by recording maximum load (*P_T_*) in terms of Equation (2):

(2) $$R_{T}=\frac{2P_{T}}{\pi Dh}(sin2\alpha -\frac{a}{D}),$$

where *h* is sample height after immersion (mm), *D* is sample diameter (mm), *α* is central angle corresponding to half width of batten (°), *a* is batten width (mm).

#### 2.3.3. Dry Shrinkage

On the basis of T0854-2009 in Chinese standard (JTG E51-2009), dry shrinkage test in Figure 4 was conducted for cuboid beam CSM samples with different substitution ratios of RCB after curing for 7 d, and the dry shrinkage coefficient could be calculated to evaluate the volume shrinkage degree of samples after water loss. Firstly, both ends of the cuboid beam CSM sample were ground flat and bonded with plexiglass sheets. Then, the cuboid beam CSM sample was located on several lubricant coated glass rods in a shrinkage instrument. Next, two dial indicators should be fastened at shrinkage device ends to contact the two ends of the cuboid beam CSM sample. The shrinkage device and samples were placed in a dry shrinkage chamber, with controlled constant conditions (20 ± 1 °C, RH 60% ± 5%). After the dry shrinkage test started, the changes in micrometer and mass data were be recorded for the tested cuboid beam samples once a day during the 1st week, and once every 2 days after that. After the dry shrinkage observation, the cuboid beam samples were dried in an oven to measure their dry mass (*m_p_*). Dry shrinkage coefficient (*α_di_*) and water loss rate (*ω_i_*) could be represented using the below equations:

(3) $$\omega_{i}=\left(m_{i}-m_{i+1}\right)/m_{p},$$

(4) $$\delta_{i}=\left(\sum_{j=1}^{4}X_{i,j}-\sum_{j=1}^{4}X_{i+1,j}\right)/2,$$

(5) $$\varepsilon_{i}={\delta_{i}/l}_{0},$$

(6) $$\alpha_{di}=\varepsilon_{i}/\omega_{i},$$

(7) $$\alpha_{d}=\sum \varepsilon_{i}/\sum \omega_{i},$$

where *m_i_* and *m_i_*_+1_ are the *i*-th and (*i* + 1)-th weighted sample mass (g), *δ_i_* is the *i*-th dry shrinkage (mm), *X_i,j_* and *X_i,j_*_+1_ are the *j*-th and (*j* + 1)-th dial indicator readings for the *i*-th observation (mm), *ε_i_* is the *i*-th dry shrinkage strain of samples (%), *l*_0_ is the length of standard samples (mm), *α_di_* is the *i*-th dry shrinkage coefficient (%) and *α_d_* is the total dry shrinkage coefficient (%).

## 3. Results and Discussion

### 3.1. Unconfined Compressive Strength Testing

#### 3.1.1. Analysis of Influence of RCB Substitution Ratios

**Figure 5:**
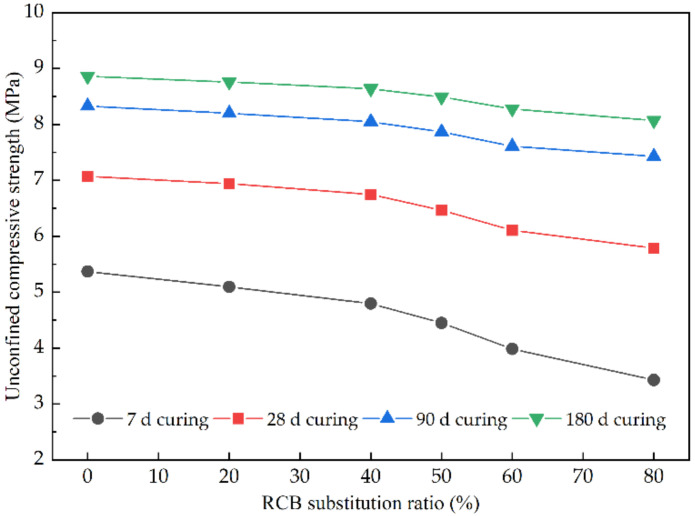
The unconfined compressive strength of CSM with different RCB substitution ratios.

Figure 5 illustrates unconfined compressive strength results for CSM containing various RCB substitution ratios at various curing time. In general, unconfined compressive strength (*R_C_*) decreases with RCB substitution rate increasing, and the *R_C_* value increases along with curing time. As shown in Figure 5, with the increase of RCB substitution ratio, the unconfined compressive strength of all CSM groups at the same curing time generally shows a downward trend, which is consistent with the conclusions in the previous studies [12,17]. Replacing BA with RCB will form a weak area inside CSM-RCB samples, resulting in a lower unconfined compressive strength of modified CSM mixtures incorporating RCB. At the same time, the unconfined compressive strength of CSM samples is related not only to the mechanical strength of aggregates, but also to the mechanical performance of interfacial transition zone. In the unconfined compression process, the failure of CSM samples generally starts from the interfacial transition zone, but the aggregate itself is rarely crushed. The RCB fine aggregates used in this study was produced by the crushing of construction waste, and the RCB angularity is not obvious compared with natural BA aggregate. Therefore, the bite force in the interfacial transition zone formed with mortar would become weaker, which is unfavorable to the unconfined compressive strength of modified CSM incorporating RCB. With the increase of RCB substitution ratio, this kind of weak interfacial transition zone will also increase inside the CSM mixtures, so the unconfined compressive strength of modified CSM incorporating RCB decreases with the increase of RCB substitution ratio.

#### 3.1.2. Analysis of Influence of Curing Time

**Figure 6:**
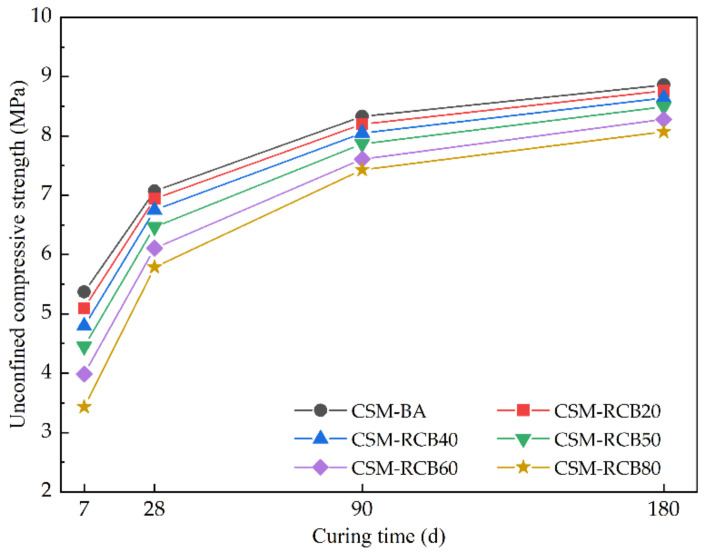
The unconfined compressive strength of CSM with different curing times.

As illustrated in Figure 6, it is worth noting that the growth law of unconfined compressive strength of CSM-RCB samples is similar to that of ordinary CSM-BA sample without RCB, and CSM samples with different RCB substitution ratios have basically the same variation trends of unconfined compressive strength. The unconfined compressive strength of all CSM groups increased sharply during the first 7 d curing period, and then its growth rate gradually slowed down from 28 d to 180 d. The growth rates of unconfined compressive strength of CSM samples with different RCB substitution ratios are also different. This may be because the hydration product by the reaction of cement and water could have wrapped the un-hydrated components, resulting in a slowdown tendency of unconfined compressive strength enhancement. In summary, most of the measurements of the early unconfined compressive strength of the CSM samples were completed after 7 d curing, and the 28 d unconfined compressive strength of CSM samples can reach about 70% of the 180 d unconfined compressive strength. These results are consistent with those of Yan’s findings [16], indicating that RCB has an obvious effect on the early unconfined compressive strength of CSM, while the negative influence of RCB on the unconfined compressive strength of CSM would decrease gradually, varying curing time. This is because that the pozzolanic reaction in CSM usually occurs at later ages after the hydration reaction of cement, and the pozzolanic reaction rate is slower than hydration reaction rate.

#### 3.1.3. Regression Analysis of Unconfined Compressive Strength

**Figure 7:**
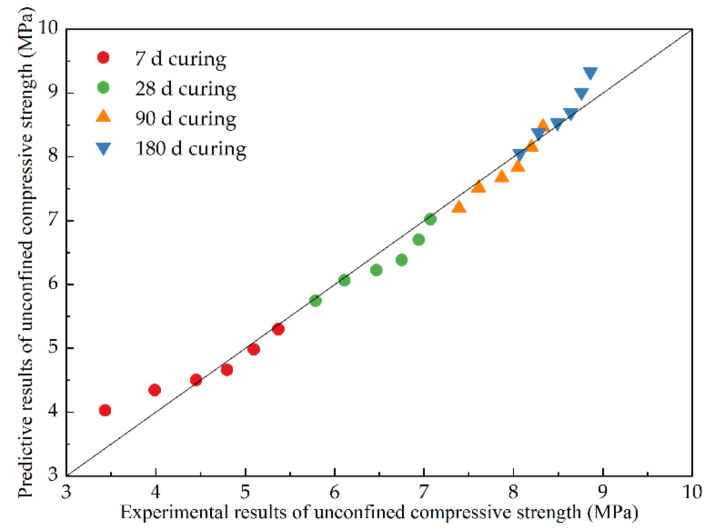
Comparison of unconfined compressive strength between experimental and predictive values.

Considering curing time and RCB substitution ratio, the unconfined compressive strength of CSM samples can be analyzed by the regression method, and the fitting equation is given by *strength* = 2.8861 + 1.2409 × ln(*x*) − 0.0159 × *y*, (R^2^ = 0.9801), where *y* is RCB substitution ratio. The fitting coefficients also indicate that the RCB substitution ratio has a negative effect on unconfined compressive strength, but the curing time has a positive effect. Figure 7 compares the experimental and predictive values of unconfined compressive strength results, which could be adopted for unconfined compressive strength prediction.

### 3.2. Indirect Tensile Strength Testing

**Figure 8:**
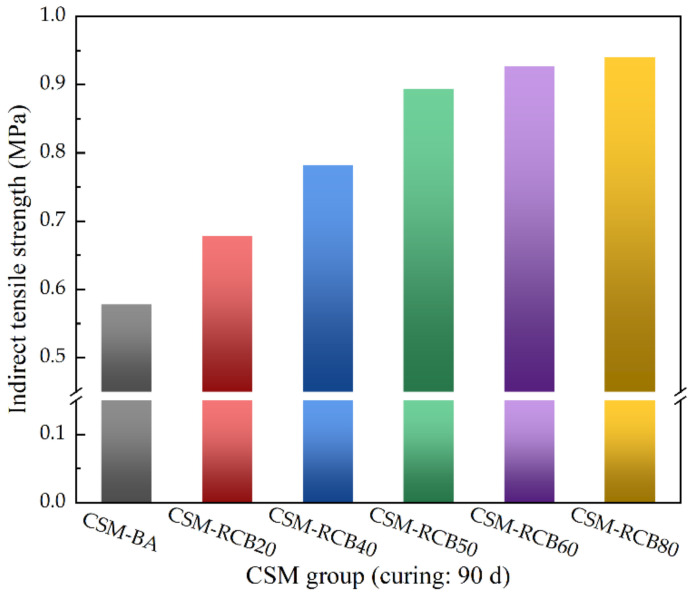
The indirect tensile strength results of CSM.

Figure 8 presents the variations of indirect tensile strength of CSM samples with different RCB substitution ratios at curing time of 90 d. As illustrated in Figure 8, as RCB substitution ratio increases, the indirect tensile strength overall presents an upward trend, which is consistent with the analysis results in the previous studies [16,29,30]. Obviously, RCB aggregate can significantly improve the indirect tensile strength of CSM samples with RCB substitution ratios from 0% to 50%; after that, the indirect tensile strength growth trend of the CSM samples becomes slow and then tends to stabilize. Compared to the CSM sample without RCB, the indirect tensile strength of CSM samples with RCB substitution ratio from 20% to 80% at curing time of 90 d increased by 17.3%, 35.3%, 54.7%, 60.4% and 62.6%, respectively. In addition, it is clearly shown that the growth speed of the indirect tensile strength for the CSM samples gradually slowed down. This result may be explained by the fact that the pozzolanic reaction usually occurs after cement hydration, and its reaction rate is relatively slow, mostly occurring during middle and late curing periods. Consequently, at 90 d curing time of the late curing period, the indirect tensile strength of modified CSM incorporating RCB would be further enhanced.

### 3.3. Dry Shrinkage Testing

#### 3.3.1. Analysis of Water Loss

**Figure 9:**
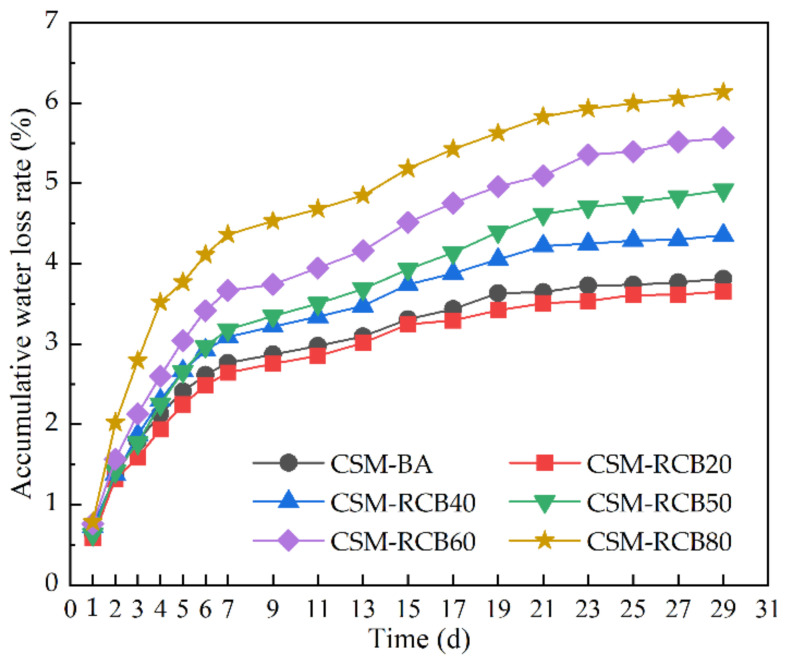
The accumulative water loss rate of CSM changing with time.

From Figure 9, the accumulative water-loss rate curve of the CSM sample incorporating 20% RCB is slightly lower compared to the ordinary CSM-BA sample without RCB. Simultaneously, the accumulative water-loss rate of CSM at the same curing time decreased first and then increased as the RCB substitution ratio increased, and the turning point is the RCB substitution ratio of 20%. This is due to the porous surface of the RCB and microcracks accompanied with the crushing production process of RCB. Furthermore, the RCB aggregate displayed a higher water absorption, so substituting the natural BA aggregate with the RCB fine aggregate would increase the OWC of the CSM samples incorporating RCB, as presented in Figure 4. It is generally considered that the water diffusivity is positively correlated with the water content (Bakhshi et al., 2012), and the larger the water content in the early curing stage of CSM samples, the more water will evaporate in the later curing stage of CSM samples, resulting in the greater dry shrinkage and water loss rate. The accumulative water loss rate of CSM-RCB80 sample at curing time of 29 d is about twice that of the ordinary CSM-BA sample without RCB. Moreover, it is clearly seen that accumulative water loss rate curves for all CSM samples are in gradually slowing upward trends with curing time increasing. The water loss rates change fast during the early curing period and gradually slow down during the later curing period.

#### 3.3.2. Analysis of Accumulative Strain of Dry Shrinkage

**Figure 10:**
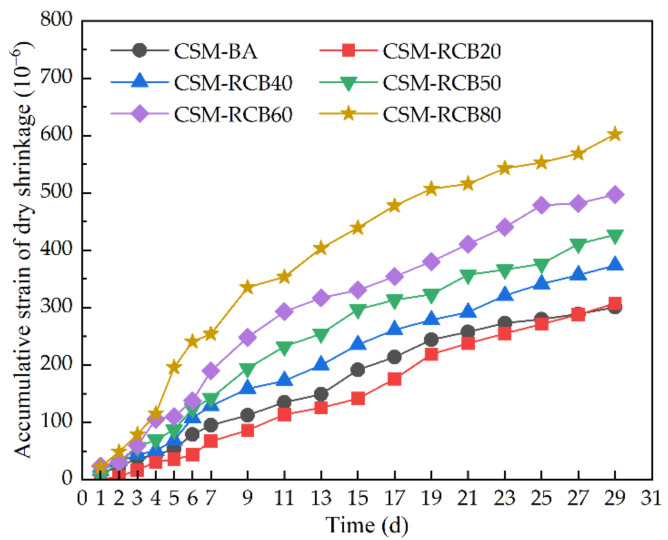
The accumulative strain of dry shrinkage changing with time.

The variations of accumulative strain of dry shrinkage changing with time are illustrated in Figure 10. As seen in Figure 10, except that the accumulative strain results of the dry shrinkage of CSM samples incorporated with 0% and 20% RCB are similar, the accumulative cumulative strain of the dry shrinkage of CSM samples at the same curing time generally increases with the increase of RCB substitution ratio, which is similar to the variations of accumulative water-loss rate. Concurrently, it should be noted that similar strain variations of dry shrinkage can always be observed for most CSM, including in this study [31], the accumulative strain of dry shrinkage increases along with curing time, and develops rapidly before a curing time of 10 d; however, the strain of dry shrinkage also displays an increasing trend at a gradually decreasing variation rate with time. Interestingly, there are similar variation trends in the accumulative water loss rate along with accumulative strain of dry shrinkage for CSM incorporating 20% RCB compared to ordinary CSM-BA without RCB, that is, the variation curves of CSM-RCB20 tend to move downward. These findings may be taken to indicate that adding an appropriate amount of RCB as a BA substitute could restrain dry shrinkage of CSM partly. Along with the increase in RCB substitution ratio range from 20% to 80%, however, there is an increasing trend of accumulative water-loss rate as well as an accumulative strain of dry shrinkage for CSM samples, since the evaporation of capillary water is significant, owing to its slow pozzolanic reaction rate. Hence, the RCB substitution ratio should be strictly controlled in practical engineering, and the water lost would attempt to compensate for the reduction of cracks caused by dry shrinkage during the semi-rigid base course conditioning period.

#### 3.3.3. Relationship Analysis between Dry Shrinkage Strain and Water-Loss Rate

**Figure 11:**
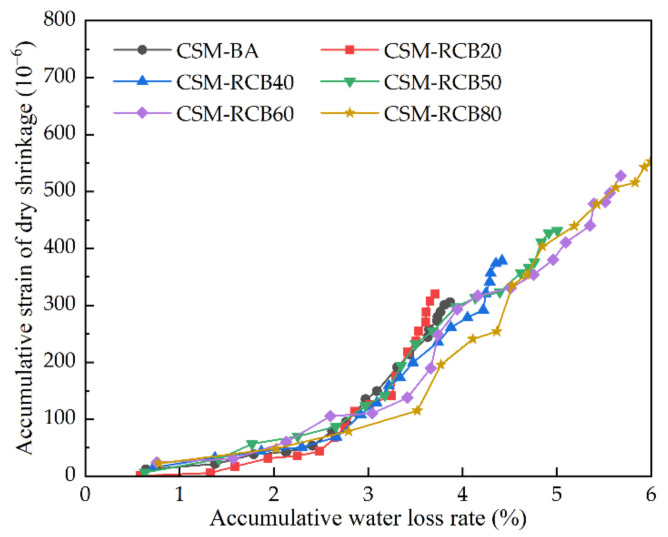
The accumulative strain of dry shrinkage changing with accumulative water loss rate.

Figure 11 provides the relationships between accumulative strain of dry shrinkage and accumulative water-loss rate among CSM samples containing different RCB substitution ratios. It can be seen from the data in Figure 11 that, with successive increases in accumulative water-loss rate, the accumulative strain of dry shrinkage of CSM samples shows a gradual rise, and the slopes of the curves incline to a rapid development. In addition, with the accumulative strain of dry shrinkage being equal, the RCB substitution ratio could increase the accumulative water-loss rate for samples, these curve slopes between accumulative strain of dry shrinkage and accumulative water-loss rate decrease with the increase of the RCB substitution ratio. These relationships may partly be explained by the lower impact of free water in macrovoids inside CSM on dry shrinkage, but the obvious impact of the dehydration of hydration products of cement and the evaporation of capillary water on dry shrinkage [32,33]. As the accumulative water-loss rate continues to grow, the evaporation of capillary water and dehydration of hydration products, which have great influence on dry shrinkage, can thereby give rise to the slope increasing.

#### 3.3.4. Analysis of Coefficient of Dry Shrinkage

**Figure 12:**
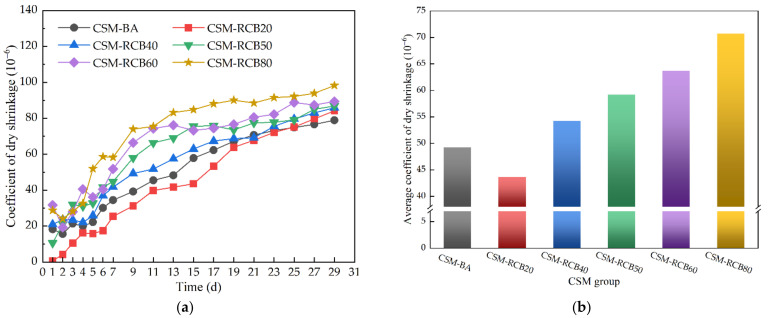
The coefficient of dry shrinkage: (**a**) coefficient of dry shrinkage with time; (**b**) average coefficient of dry shrinkage.

Generally, the coefficient of dry shrinkage is used to reflect the sensitivity of base course materials to water, that is, the sensitivity coefficient. The more sensitive to water the base course material is, the greater its coefficient of dry shrinkage, indicating a worse crack resistance. Figure 12 provides the coefficient of dry shrinkage and average coefficient of dry shrinkage, respectively. As can be seen, the dry shrinkage coefficient of total CSM samples increases gradually as curing age increases, and the dry shrinkage curve is likely to remain steady after a longer curing time. By contrast, because of the larger water absorption of RCB, increasing RCB substitution ratio in aggregates can result in a larger coefficient of dry shrinkage. The average coefficient of dry shrinkage of CSM sample incorporating RCB of 80% is 43.5% higher than that of ordinary CSM-BA sample without RCB. Moreover, the coefficient of dry shrinkage first decreases and then increases, and its minimum value appears at the RCB substitution ratio of 20%, and the average coefficient of dry shrinkage of CSM-RCB20 is less than that of CSM-BA. This result may be explained by the fact that the water loss of macrovoids inside CSM samples has no obvious influence on dry shrinkage.

As discussed above, considering the porous surface of RCB and its higher water absorption, the porosity of CSM samples would be elevated, and the water content is generally required for CSM material compaction. Meanwhile, as an important part of the CSM base construction, curing directly affects the final strength of base course and the formation of potential cracks, which should be paid great attention to. During the curing period of base course, it is necessary to frequently spray water to prevent the rapid loss of water on the surface from causing dry shrinkage cracks, and ensure that sufficient water is given to meet the needs of cement hydration. If possible, a geomembrane or geotextile can be used to cover to reduce the volatilization of water and reduce the number of spraying water. In actual pavement works, according to Chinese standard (JTG E51-2009), the semi-rigid base material is usually required to be cured for 7 d after construction to reach a certain strength.

### 3.4. Strength Mechanism Analysis

**Figure 13:**
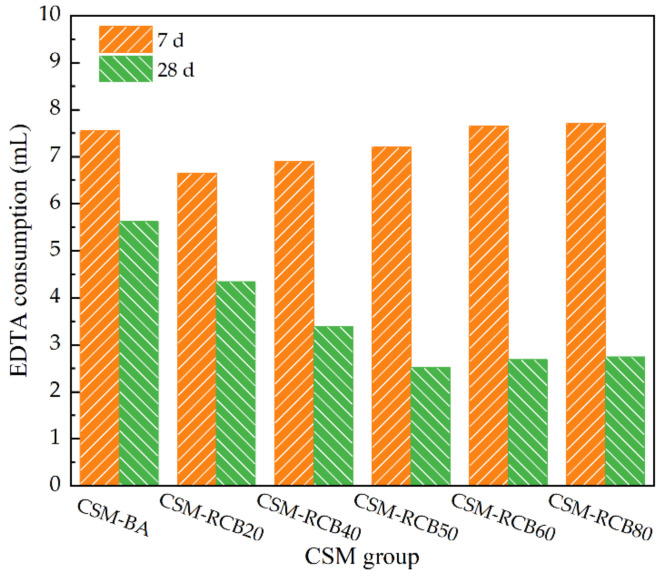
The EDTA consumption with curing for CSM samples.

For a deep understanding of the strength mechanism of modified CSM incorporating RCB intuitively, chemical detection methods have been used to test the pozzolanic activity. The EDTA titration method has been extensively applied as an effective means to check the cement content of CSM. The Ca^2+^ from the hydration product could be considered to be the same for all CSM samples due to the cement dosage in mixture, while the Ca^2+^ provided by the RCB aggregates has a positive correlation with the RCB substitution ratio. In the meantime, the pozzolanic materials in RCB aggregates continuously react with the hydration product (calcium hydroxide) to generate hydrated calcium silicate (C-S-H), which is insoluble in water, reducing the concentration of Ca^2+^. The ethylenediaminetetraacetic acid disodium (EDTA-2Na) is a chelating agent with six coordination atoms, which can combine with metal ions such as Ca^2+^ and Mg^2+^ to produce the complexes with cyclic structure due to the chelation [34]. From Figure 13, it should be noted that modified CSM containing pozzolanic components reacts slowly, divalent cation consumption as Ca^2+^ mainly occurs at later ages. These findings could prove the pozzolanic material of RCB aggregates, and the pozzolanic reaction rate is slower than hydration reaction rate. The continuous pozzolanic reaction will form the crystal structure, cementing into a whole and enhancing the interfacial transition zone, which has a positive effect explained from chemical reaction on the strength of the modified CSM incorporating RCB.

## 4. Conclusions

The reutilization of RCB in CSM is a promising solution for C&D waste disposal. The present work evaluates the feasibility of CSM incorporating various RCB fine aggregate substitution ratios ranging from 0% to 80%. The experimental results and discussions lead to the following conclusions:

(1) The negative influence of RCB on the unconfined compressive strength of CSM would decrease gradually, varying the curing time due to a slower pozzolanic reaction rate. In contrast, the higher the RCB substitution ratio, the larger the indirect tensile strength at 90 d curing time of the late curing period.
(2) Substituting RCB for natural aggregate resulted in an overall increasing accumulative water-loss rate, accumulative strain of dry shrinkage and average coefficient of dry shrinkage, except for the CSM with 20% RCB (i.e., CSM-RCB20), which obtained an excellent dry shrinkage property, and the average coefficient of dry shrinkage reached the minimum value.
(3) CSM incorporating RCB with pozzolanic activity reacts very slowly to form the crystal structure, cementing into a whole and enhancing the interfacial transition zone, which has a positive effect on the strength of modified CSM mainly at later ages.

The present study demonstrated the primary feasibility of incorporating recycled crushed clay brick from C&D waste as fine aggregate into cement-stabilized macadam of pavement base, which would be a sustainable means for achieving environmental protection and resource conservation, and even reducing the carbon footprint of raw materials mining. However, more research is needed, including in-depth comprehensive performances, economic assessment and sustainability evaluation for deeper investigation into the practical applications of CSM with RCB, which is a tough work. Moreover, the possibility of the entrance of rainwater via pavement cracks is impossible to avoid, and the layer position and performance of CSM incorporating RCB should be also considered in the future studies.

## Figures and Tables

**Table 1:** The technical indicators of BA and RCB aggregates.

Aggregates	Apparent Specific Density	Water Absorption (%)	Flakiness Content (%)	Crushed Stone Value (%)	Liquid Limit (%)	Plasticity Index
Coarse BA	2.735	1.24	9.57	21.63	/	/
Fine BA	2.689	1.73	/	/	18.06	4.34
Coarse RCB	2.331	17.36	9.97	41.53	/	/
Fine RCB	2.116	17.60	/	/	37.91	8.50

## Data Availability

The testing and analysis data used to support the findings of this study are included within the article.

## References

[1] Wong C.L., Mo K.H., Yap S.P., Alengaram U.J., Ling T.C. (2018). Potential use of brick waste as alternate concrete-making materials: A review. J. Clean. Prod..

[2] Arulrajah A., Piratheepan J., Disfani M.M., Bo M.W. (2013). Geotechnical and geoenvironmental properties of recycled construction and demolition materials in pavement subbase applications. J. Mater. Civ. Eng..

[3] Pappu A., Saxena M., Asolekar S.R. (2007). Solid wastes generation in india and their recycling potential in building materials. Build. Environ..

[4] Wu J.D., Guo L.P., Qin Y.Y. (2021). Preparation and characterization of ultra-high-strength and ultra-high-ductility cementitious composites incorporating waste clay brick powder. J. Clean. Prod..

[5] Duan H.B., Li J.H. (2016). Construction and demolition waste management: China’s lessons. Waste Manag. Res..

[6] Gebremariam A.T., Vahidi A., Di Maio F., Moreno-Juez J., Vegas-Ramiro I., Lagosz A., Mroz R., Rem P. (2021). Comprehensive study on the most sustainable concrete design made of recycled concrete, glass and mineral wool from C&D wastes. Constr. Build. Mater..

[7] Tang D.W., Zhang X.B., Hu S.S., Liu X.Y., Ren X., Hu J.X., Feng Y. (2020). The reuse of red brick powder as a filler in styrene-butadiene rubber. J. Clean. Prod..

[8] Xuan D.X., Molenaar A.A.A., Houben L.J.M. (2012). Compressive and indirect tensile strengths of cement-treated mix granulates with recycled masonry and concrete aggregates. J. Mater. Civ. Eng..

[9] Li L.G., Lin Z.H., Chen G.M., Kwan A.K.H., Li Z.H. (2019). Reutilization of clay brick waste in mortar: Paste replacement versus cement replacement. J. Mater. Civ. Eng..

[10] Afshinnia K., Poursaee A. (2015). The potential of ground clay brick to mitigate alkali-silica reaction in mortar prepared with highly reactive aggregate. Constr. Build. Mater..

[11] Yang J.A., Du Q.A., Bao Y.W. (2011). Concrete with recycled concrete aggregate and crushed clay bricks. Constr. Build. Mater..

[12] Yan K.Z., Li G.K., You L.Y., Zhou Y.B., Wu S.H. (2020). Performance assessments of open-graded cement stabilized macadam containing recycled aggregate. Constr. Build. Mater..

[13] Sun Y., Li L.H. (2018). Strength assessment and mechanism analysis of cement stabilized reclaimed lime-fly ash macadam. Constr. Build. Mater..

[14] Chhabra R.S., Ransinchung G.D.R.N., Islam S.S. (2021). Performance analysis of cement treated base layer by incorporating reclaimed asphalt pavement material and chemical stabilizer. Constr. Build. Mater..

[15] Zhao C., Liang N., Zhu X., Yuan L., Zhou B. (2020). Fiber-reinforced cement-stabilized macadam with various polyvinyl alcohol fiber contents and lengths. J. Mater. Civ. Eng..

[16] Yan K.Z., Sun H., Gao F.Q., Ge D.D., You L.Y. (2020). Assessment and mechanism analysis of municipal solid waste incineration bottom ash as aggregate in cement stabilized macadam. J. Clean. Prod..

[17] Zhang J.H., Li C., Ding L., Li J. (2021). Performance evaluation of cement stabilized recycled mixture with recycled concrete aggregate and crushed brick. Constr. Build. Mater..

[18] Arulrajah A., Piratheepan J., Bo M.W., Sivakugan N. (2012). Geotechnical characteristics of recycled crushed brick blends for pavement sub-base applications. Can. Geotech. J..

[19] Arabani M., Moghadas Nejad F., Azarhoosh A.R. (2013). Laboratory evaluation of recycled waste concrete into asphalt mixtures. Int. J. Pavement Eng..

[20] Zhao Z., Wang S.Y., Ren J.L., Wang Y., Wang C.G. (2021). Fatigue characteristics and prediction of cement-stabilized cold recycled mixture with road-milling materials considering recycled aggregate composition. Constr. Build. Mater..

[21] Poon C.S., Chan D.X. (2006). Paving blocks made with recycled concrete aggregate and crushed clay brick. Constr. Build. Mater..

[22] Adamson M., Razmjoo A., Poursaee A. (2015). Durability of concrete incorporating crushed brick as coarse aggregate. Constr. Build. Mater..

[23] Bektas F., Wang K., Ceylan H. (2009). Effects of crushed clay brick aggregate on mortar durability. Constr. Build. Mater..

[24] Khatib J.M. (2005). Properties of concrete incorporating fine recycled aggregate. Cem. Concr. Res..

[25] Shao J., Gao J., Zhao Y., Chen X. (2019). Study on the pozzolanic reaction of clay brick powder in blended cement pastes. Constr. Build. Mater..

[26] Li Q., Wang Z., Li Y., Shang J. (2018). Cold recycling of lime-fly ash stabilized macadam mixtures as pavement bases and subbases. Constr. Build. Mater..

[27] Ortega J.M., Letelier V., Solas C., Moriconi G., Climent M.Á., Sánchez I. (2018). Long-term effects of waste brick powder addition in the microstructure and service properties of mortars. Constr. Build. Mater..

[28] Liang C.Y., Wang Y., Song W.Z., Tan G.J., Li Y.L., Guo Y.M. (2019). Potential activity of recycled clay brick in cement stabilized subbase. Appl. Sci..

[29] Yan K., Gao F., Sun H., Ge D., Yang S. (2019). Effects of municipal solid waste incineration fly ash on the characterization of cement-stabilized macadam. Constr. Build. Mater..

[30] Raavi S.S.D., Tripura D.D. (2020). Predicting and evaluating the engineering properties of unstabilized and cement stabilized fibre reinforced rammed earth blocks. Constr. Build. Mater..

[31] Zhao G., She W., Yang G., Pan L., Cai D., Jiang J., Hu H. (2017). Mechanism of cement on the performance of cement stabilized aggregate for high speed railway roadbed. Constr. Build. Mater..

[32] Fan S., Wang P. (2017). Effect of fly ash on drying shrinkage of thermal insulation mortar with glazed hollow beads. J. Wuhan Univ. Technol.-Mater. Sci. Ed..

[33] Yang J., Liu L., Liao Q., Wu J., Li J., Zhang L. (2019). Effect of superabsorbent polymers on the drying and autogenous shrinkage properties of self-leveling mortar. Constr. Build. Mater..

[34] Yan X., Jiang L., Guo M., Chen Y., Zhu P., Jin W., Zha J. (2020). Using edta-2na to inhibit sulfate attack in slag cement mortar under steam curing. Constr. Build. Mater..

